# Thermal-Induced Performance Decay of the State-of-the-Art Polymer: Non-Fullerene Solar Cells and the Method of Suppression

**DOI:** 10.3390/molecules28196856

**Published:** 2023-09-28

**Authors:** Xingxing Qin, Xuelai Yu, Zerui Li, Jin Fang, Lingpeng Yan, Na Wu, Mathias Nyman, Ronald Österbacka, Rong Huang, Zhiyun Li, Chang-Qi Ma

**Affiliations:** 1 i-Lab &Printed Electronics Research Center, Suzhou Institute of Nano-Tech and Nano-Bionics, Chinese Academy of Sciences (CAS), Suzhou 215123, Chinaronald.osterbacka@abo.fi (R.Ö.); 2 Nano Science and Technology Institute, University of Science and Technology of China, 166 Ren Ai Road, SEID SIP, Suzhou 215123, China; 3 School of Nano-Tech and Nano-Bionics, University of Science and Technology of China, 398 Jinzhai Road, Hefei 230026, China; 4 College of Materials Science and Engineering, Taiyuan University of Technology, Taiyuan 030024, China; 5 Physics and Center for Functional Materials, Faculty of Science and Technology, Åbo Akademi University, Henriksgatan 2, 20500 Turku, Finland; mathias.nyman@abo.fi; 6 Vacuum Interconnected Nanotech Workstation, Suzhou Institute of Nano-Tech and Nano-Bionics, Chinese Academy of Sciences (CAS), 398 Ruoshui Road, SEID, SIP, Suzhou 215123, China

**Keywords:** non-fullerene solar cells, thermal degradation, C_60_, surface passivation, stability improvement

## Abstract

Improving thermal stability is of great importance for the industrialization of polymer solar cells (PSC). In this paper, we systematically investigated the high-temperature thermal annealing effect on the device performance of the state-of-the-art polymer:non-fullerene (PM6:Y6) solar cells with an inverted structure. Results revealed that the overall performance decay (19% decrease) was mainly due to the fast open-circuit voltage (*V*_OC_, 10% decrease) and fill factor (FF, 10% decrease) decays whereas short circuit current (*J_SC_*) was relatively stable upon annealing at 150 °C (0.5% decrease). Pre-annealing on the ZnO/PM6:Y6 at 150 °C before the completion of cell fabrication resulted in a 1.7% performance decrease, while annealing on the ZnO/PM6:Y6/MoO_3_ films led to a 10.5% performance decay, indicating that the degradation at the PM6:Y6/MoO_3_ interface is the main reason for the overall performance decay. The increased ideality factor and reduced built-in potential confirmed by dark *J* − *V* curve analysis further confirmed the increased interfacial charge recombination after thermal annealing. The interaction of PM6:Y6 and MoO_3_ was proved by UV-Vis absorption and XPS measurements. Such deep chemical doping of PM6:Y6 led to unfavorable band alignment at the interface, which led to increased surface charge recombination and reduced built-in potential of the cells after thermal annealing. Inserting a thin C_60_ layer between the PM6:Y6 and MoO_3_ significantly improved the cells’ thermal stability, and less than 2% decay was measured for the optimized cell with 3 nm C_60_.

## 1. Introduction

In recent years, non-fullerene acceptor (NFA) molecules for use in polymer solar cells have attracted vast research interest due to their better absorption ability, variety in molecular design, adjustable energy levels and excellent compatibility with polymer donor materials [1,2,3]. At present, the highest power conversion efficiencies (PCE) of 19.6% were reported for a ternary polymer:NFA single-junction solar cell [4], bringing this type of solar cell closer to commercialization. Compared with constantly improved efficiency, the device stability is also significant, where various factors such as light, heat, oxygen and moisture could cause the fast performance decrease. Among them, photo-induced degradation processes of polymer:NFA solar cells were investigated most, and some degradation pathways were gradually clarified in the last few years. For example, the interfacial degradation between ZnO and organic BHJ was found to be the key factor causing poor performance stability, where Zhou and Park et al. proved that UV light illumination would cause the decomposition of NFA molecules on ZnO, consequently decreasing device performance [5,6]. Lee et al. reported that the reoxidation of a ZnO ETL resulting from slow diffusion of ambient oxygen or water lowered the electron quasi-Fermi level and increased the energy barrier at the ZnO-Active layer junction [7]. Our recent work further proved that the oxidation of the dangling hydroxyl group of the ZnO surface causes interfacial photodecomposition of NFA molecules by forming chemical reactive hydroxy radicals (OH·) with light illumination, which can be suppressed by proper surface passivation [8]. By proper interfacial protection of the ZnO surface with Lewis acid, such as 2-phenylethanethiol, zirconium acetylacetonate or glucose, high-performance inverted PM6:Y6 cells with an initial PCE of over 16% showed that a log *T*_80_ of over 4000 h was achieved [9]. By using L8-BO, an NFA with a branched alkyl side chain on the β-position of the thiophene unit next to the C=C bond of the terminal group, a solar cell with PCE of 17% showed a *T*_80_ of over 5000 h [10], which is among the most stable polymer solar cells with a high initial device performance [11,12,13].

Aside from photostability, the thermal stability of polymer solar cells is also crucial for their industrialization. At least two essential scenarios require high thermal stability for polymer solar cells. Under actual operational conditions, solar cells can suffer from high temperatures (up to 80 °C) [14,15,16]. For the fabrication of solar modules, cover encapsulation with glass or barrier film is quite commonly used geometry, and hot-pressing encapsulation (over 150 °C) is a key process [17,18]. Since the performance of polymer solar cells is highly dependent on the nanostructure of the photoactive layer, the studies of the thermal stability of polymer:NFA solar cells mainly focus on the influence of thermal annealing on the morphology of the photoactive layer. For example, Song et al. reported that J52:i-IEICO-2F devices, with almost identical morphology and molecular orientation under thermal annealing at 150 °C, directly correlated to the PCE stability against thermal annealing [19]. For the morphology instability during thermal annealing, Müller et al. reported that the crystallization processes would be much different with the temperature increasing [20]. Min et al. reported that in a PM6:BTTT-2Cl-based device, the homogeneity of BHJ blend was altered after heating, and many long string-like BTTT-2Cl crystals were observed in the aged blend [21]. These studies proved that the morphology of the active layer would change under high temperatures, which is highly related to the device performance. Correspondingly, various additives have been introduced to improve the device’s thermal stability through enhancing morphology stability, such as indium selenide (In_2_Se_3_) nanosheets [22], polymer [21] and resin [23]. In addition to the photoactive layer, interfacial degradation under thermal annealing between ZnO and BHJ was also reported to cause the morphology change of the active layer [24]. However, device performance still exhibited degradation under thermal stress, indicating that other degradation pathways still be hidden behind. Studying the detailed thermal degradation mechanism and finding corresponding solutions is a significant issue. Even though only few papers report the thermal degradation behaviors and mechanism of high-efficiency NFA solar cells, much less is known about the detailed degradation mechanism, especially on BHJ/MoO_3_ interface. There is no commonality among materials systems, and the literature reports are rare on exploring methods for stability improvement.

For interfacial degradation, although photo-induced decomposition was reported for both fullerene and non-fullerene cells [5,6,7,25], not many research works were put onto thermal-induced interfacial degradation [26,27]. As demonstrated later in this article, thermal-induced interfacial degradation is the most prominent degradation pathway for the inverted PM6:Y6 cells. By comparing the performance decay rate of the cells with different pre-annealed films, we confirmed that the PM6:Y6/MoO_3_ interface is responsible for the interfacial decay. Increased interfacial recombination and decreased built-in potential of the cells were confirmed to be the physical reasons for the thermal-induced performance decay while the chemical doping of PM6:Y6 by MoO_3_ is the chemical reason. Inserting a thin n-type C_60_ layer between PM6:Y6 and MoO_3_ partially improved the thermal stability of the cells. For the first time, the current work revealed the mechanism of thermal-induced interfacial degradation of NFA solar cells and explored an effective way to solve the problem, which is expected to promote the commercial application of organic solar cells.

## 2. Results and Discussion

### 2.1. Performance and Thermal Stability of the PM6:Y6 Cells

**Figure 1:**
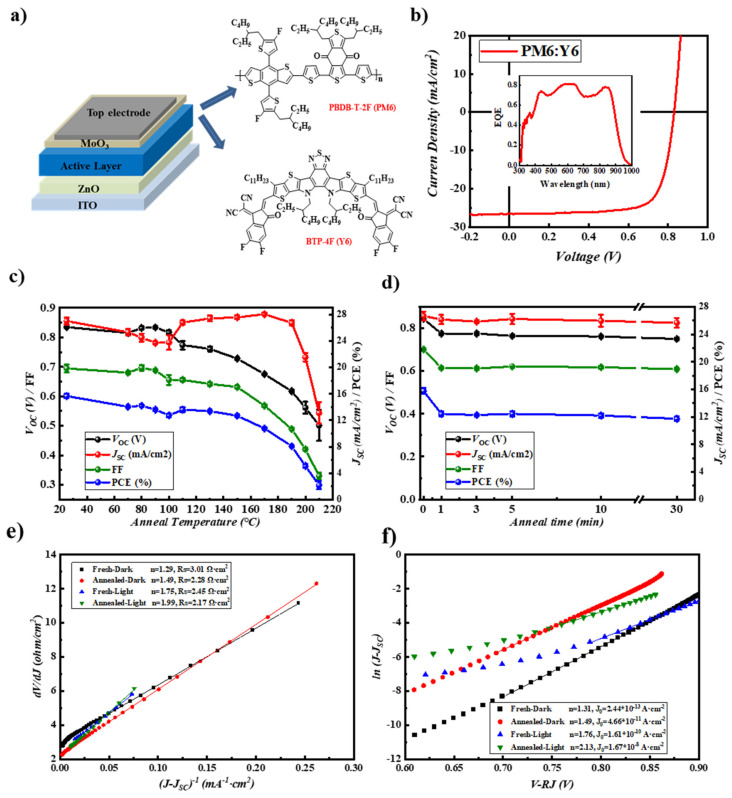
(**a**) Schematic illustration of the device structure and chemical structures of materials used in this work; (**b**) the representative *J* − *V* curve and EQE curve of PM6:Y6 devices; (**c**) the device performance variation of PM6:Y6 devices annealed at different temperatures for 30 min; (**d**) performance parameters of PM6:Y6 devices annealed at 150 °C for different times. (**e**,**f**) The fitted curves of *dV/dJ* vs. (*J* − *J_SC_*)^−1^ and ln(*J* − *J_SC_*) vs. *V* − *R_s_J* for the pristine and aged cells annealed at 150 °C for 30 min.

Inverted polymer solar cells with a structure of ITO/ZnO/active layer/MoO_3_/Al (Figure 1a) using PM6:Y6 blend as the photoactive layer were fabricated. Figure 1b shows the *J* − *V* curve and EQE spectrum of the best-performed cell, which showed a *V*_OC_ of 0.831 V, a *J_SC_* of 26.56 mA/cm^2^, an FF of 0.75 and an overall PCE of 16.53%. As seen here, the prepared PM6:Y6 cells showed an averaged PCE of 16.19% ± 0.18%, comparable to that reported in the literature with an inverted device structure [24,28,29], indicating that all these cells were well optimized. The cells were then thermally annealed at different temperatures for 30 min, upon which the evolution of photovoltaic performance was shown in Figure 1c (from 70 °C to 210 °C). Figure S1a in supporting information shows the *J* − *V* curves of representative cells after being annealed at different temperatures. Although there is a slight efficiency increase between 80 to 100 °C, the PCE of the cells decreases gradually with the increasing annealing temperatures, mainly due to the decrease of *V*_OC_ and FF [30]. Interestingly, *J_SC_* increases significantly when the cells are annealed at 110 °C and then remains at a high value of 26–27 mA/cm^2^ even at an annealing temperature of 170 °C. Further increase of the annealing temperature to 190–210 °C decreases the *J_SC_* significantly, yielding a significant PCE decay. All the data for these devices are listed in Table S1 in supporting information.

We then specifically investigated the performance decay of the PM6:Y6 cells annealed at 150 °C, which is mainly used in hot-press encapsulation [31]. Figure 1d shows the evolution of the device performance upon different annealing times, and Figure S1b in supporting information shows the *J* − *V* curves of the representative cells. As seen here, *V*_OC_ and FF decayed by over 10% at the first annealing minute and then became rather stable with a further increase in the annealing time. In contrast, *J_SC_* was relatively stable over a long annealing time. All the data for these devices were listed in Table S2 in supporting information.

*J* − *V* characteristic of a single junction solar cell is described with one diode model as the following [32,33,34]:

(1) $$J=J_{0}\left\{exp\left[\frac{q\left(V-JR_{sh}\right)}{nk_{B}T}\right]-1\right\}+\frac{V-JR_{s}}{R_{sh}}+J_{SC}$$

where *J*_0_, *q*, *n*, *R_s_*, *R_sh_* represent the reverse saturated current density, elementary charge, the ideal factor of the diode, the series resistance and shunt resistance of PSCs while *k_B_* and *T* represent the Boltzmann constant and absolute temperature, respectively. Equation (1) can be transferred to the following two Equations (2) and (3):

(2) $$\frac{dV}{dJ}=\frac{nK_{B}T}{q}{\left(J-J_{SC}\right)}^{-1}+R_{S}$$

(3) $$\ln\left(J-J_{SC}\right)=\frac{q}{nK_{B}T}\left(V-R_{S}J\right)+lnJ_{0}$$

Figure 1e depicts the plots of *dV*/*dJ* vs. (*J* − *J_SC_*)^−1^ for the pristine and aged cells annealed at 150 °C for 30 min. The linear fitting curves for each cell according to Equation (2) were also shown in this figure. The R_S_ and n of the cells were then obtained from the intercept and slope of the linear fitting curves, which are listed in these figures. As seen here, upon thermal annealing, RS decreases slightly from 2.45 to 2.17 Ω/cm^2^ (under light illumination) and from 3.01 to 2.08 Ω/cm^2^ (in the dark) whereas the ideality factor (*n*) increases from 1.75 to 1.99 (under light illumination) and from 1.29 to 1.49 (in the dark). The decreased *R_S_* indicates increased charge extraction while the increased n indicates the increased trap-assisted recombination upon thermal annealing. The plots of ln(*J* − *J_SC_*) vs. (*V* − *R_S_J*) of these cells are shown in Figure 1f, and a linear relationship was also found for these cells in the space charge region. The ideality factor (*n*) and reverse saturated current density (*J*_0_) were then derived from the linear fitting results. The *n* values for the pristine and annealed cells were calculated to be very close to that derived from *dV*/*dJ* vs. (*J* − *J_SC_*)^−1^ curves (Figure 1e). The cells’ reverse saturated current density (*J*_0_) increased after annealing, which can also be ascribed to the increased surface recombination within the cell after thermal annealing.

To understand the influence of thermal annealing on the photoactive layer, hole-only devices with a structure of ITO/PEDOT:PSS/PM6:Y6/MoO_3_/Al and electron-only devices with a structure of ITO/ZnO/PM6:Y6/C_60_/Al were fabricated and tested. The *J − V* curves for these devices are shown in Figure S2, and the charge carrier mobility of the photoactive layer was obtained by fitting the *J* − *V* results to the Mott Equation (4):

(4) $$J=\frac{9\varepsilon_{0\;}\varepsilon_{r}\mu V^{2}}{8L^{3}}$$

where *J* is the current density, *ε*_0_ is the permittivity of free space (8.85 × 10^−12^ F/m), *ε_r_* is the dielectric constant of the organic blends, which was assumed to be 3.5 for PM6:Y6, *μ* is the electron mobility, *V* is the voltage drop across the device and *L* is the thickness of the active layer. Thermal annealing at 150 °C increases the hole mobilities (*μ_h_*) of the photoactive layer from 6.56 × 10^−5^ to 2.94 × 10^−4^ cm^2^/V·s and the electron mobilities (*μ_e_*) of the photoactive layer from 1.63 × 10^−5^ to 2.99 × 10^−5^ cm^2^/V·s, respectively, indicating that the decrease of solar cell performance upon thermal annealing should not be ascribed to the change of the photoactive layer. Almost no change was observed in AFM images of PM6:Y6 films (shown in Figure S3), further proving that such a thermal-induced degradation does not come from the morphology change of the photoactive layer.

### 2.2. Identifying the Interface Degradation of the Cells

**Figure 2:**
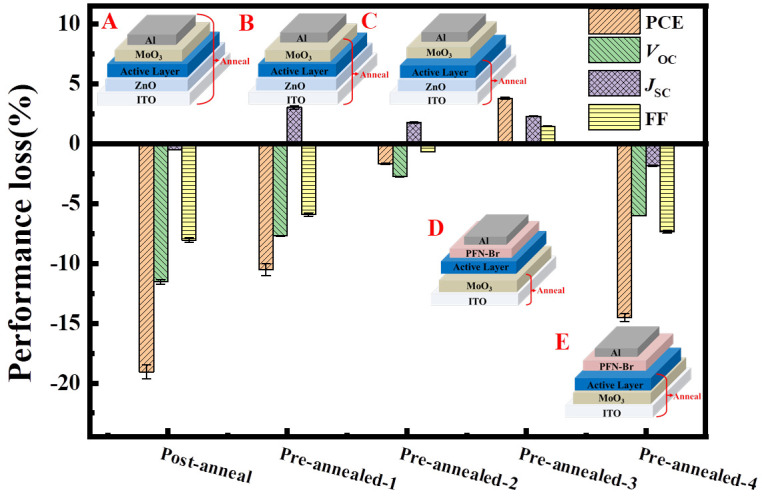
Comparison of the performance loss of the cells with different annealing on the functional layers at 150 °C for 30 min.

To identify which interface or layer causes the performance degradation upon thermal annealing, pre-annealing on different half-cells, including ITO/ZnO/PM6:Y6 (Device A, standard cell), ITO/ZnO/PM6:Y6/MoO_3_ (Device B) and ITO/ZnO/PM6:Y6 (Device C) at 150 °C for 30 min prior to the completion of cell fabrication were carried out. After the completion of the cell, post-annealing at 150 °C for 30 min was performed on these cells. The cell performances were checked before and after thermal annealing. Figure 2 shows the performance variation for these cells after post-thermal annealing. The complete data are listed in Figures S4 and S5 and Tables S4–S6. As seen here, Device A (standard device) shows an almost unchanged *J_SC_* (0.5% decrease) but obvious *V*_OC_ (−12%) and FF (−8%) decay and an overall PCE decrease of 19%. Device B showed a similar PCE decay (−11%) with fast *V*_OC_ (−8%) and FF (−12%) decays but a slightly increased *J_SC_* (3%). On the contrary, Device C showed much fewer performance variations (with −3% for *V*_OC_, +2% for *J_SC_*, −0.7% for FF and −1.7% for PCE). These results demonstrated that thermal annealing-induced performance decay of PM6:Y6 cells is mostly originated from the decay at PM6:Y6/MoO_3_, which could be due to the decomposition of MoO_3_ itself and/or the decay of the PM6:Y6/MoO_3_ interface.

We then compared the influence of pre-annealing on the device performance of the ITO/MoO_3_ (Device D) and ITO/MoO_3_/PM6:Y6 films (Device E) prior to the completion of the conventional cells with a structure of ITO/MoO_3_/PM6:Y6/PFN-Br/Ag. The results are also shown in Figure 2, Figure S6 and Table S7 as well. As seen here, Device D showed a slight increase of device performance upon thermal annealing, owing to the increase of *J_SC_* and FF (no noticable change for *V*_OC_), while Device E led to obvious *V*_OC_ (−6%) and FF (−7%) decays and an overall 15% PCE decay. The measured performance decay of device E confirmed that the thermal-induced performance decay of PM6:Y6 cells is primarily due to the decay of PM6:Y6/MoO_3_ interface rather than the decomposition of MoO_3_ itself. In addition, we renewed the MoO_3_/Al top electrode according to the method reported previously [35]. Figure S7 shows the *J* − *V* curves of the aged cell before and after renewing the electrode. Results showed that *V*_OC_ and FF were partially recovered after renewing the top electrode, further confirming that decomposition at PM6:Y6/MoO_3_ interface could be the main reason for the performance decay of the cells.

### 2.3. Interactions at the Polymer/MoO_3_ Interface upon Thermal Annealing

**Figure 3:**
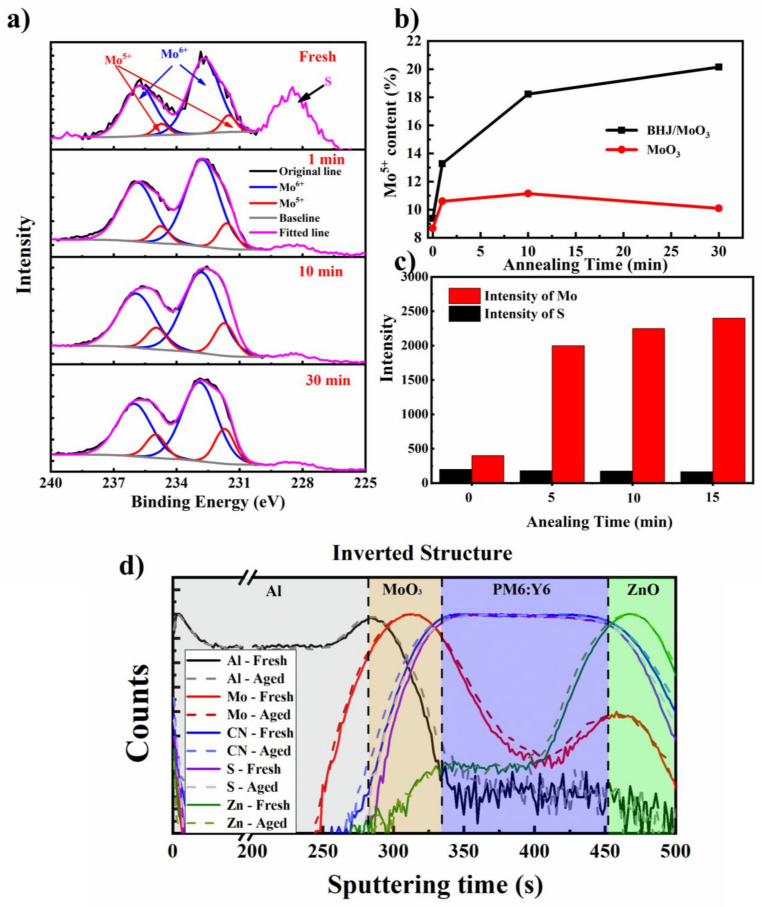
(**a**) XPS spectra of the Al/MoO_3_ surface peeled from the cells after thermal annealed for different times; (**b**) change of Mo^5+^/(Mo^5+^ + Mo^6+^) of the Al/MoO_3_ surface with different annealing times. The change of Mo^5+^ content of the pure MoO_3_ film upon thermal annealing was also included for comparison; (**c**) XPS signal intensity of Mo 3d and S 2s, which shows the almost identical S 2s intensity and increased Mo 3d intensity upon thermal annealing; (**d**) SIMS element distribution over the cell before and after thermal annealing for 30 min at 150 °C.

Our previous works demonstrated that the interface photon reduction of MoO_3_ with organic molecules inevitably happens, which causes fast *V*_OC_ and FF decays [36]. We checked the chemical state of Mo at the interface by measuring the XPS of the surface of the peeled electrode from the cells. Figure 3a shows the high-resolution XPS spectra of the MoO_3_ surface at an energy range of 225–240 eV. As seen here, both S 2s (228.5 eV) and Mo 3d (231–238 eV) signals were measured, indicating the successful peeling off of the Al/MoO_3_ electrode from the photoactive layer, similar to that reported in the literatures [26,37,38,39]. The XPS spectra of Mo 3d were fitted by two groups of peaks, Mo^6+^ (235.9 and 232.8 eV) and Mo^5+^ (234.9 and 231.7 eV) [40]. Proportion ratios of Mo^5+^/Mo^6+^ for these films were then calculated by comparing the integrated area for each set of peaks, and the results are listed in Tables S11 and S12. As seen here, with the increase of thermal annealing time, the Mo^5+^/Mo^6+^ ratio increased from 9.4% to 20.15%, which is much higher than the pristine MoO_3_ film (Figure 3b), indicating that Mo^6+^ was partially reduced upon thermal annealing on the top of PM6:Y6. Based on these results, forming an intermolecular charge transfer complex between MoO_3_ and conjugated polymers promoted by thermal heating was proposed to be the main chemical reason for the interfacial decay, similar to that reported by Gunther et al. [26,40]. Interestingly, we found that the intensity of the S 2s signal is almost identical upon thermal annealing while the Mo signal intensity increases significantly under thermal annealing. Knowing that the detection depth of XPS is usually around 5–7 nm, the increased MoO_3_ signal indicates a significant inter-diffusion of MoO_3_ and the organic layer, which was also supported by the increased C\O signals (Figures S9–S11, Tables S9 and S10).

We then checked the element distribution of the cell before and after thermal annealing with TOF-SIMS. The results are shown in Figure 3d. The distribution of Y6 was estimated from the CN profile since only molecule Y6 has N atoms. Meanwhile, we use the S profile to estimate the distribution of PM6 over the cell since the S ratio of PM6 is almost 1.5 times that of Y6. As seen here, layer boundaries can be clearly distinct from the SIMS results. The metal elements showed almost no change upon thermal annealing, which is different from that reported by Andersson et al., where diffusion of Mo into 5–6 nm P3HT:PC_61_BM layer was confirmed after annealing 170 °C for 10 min [26]. The lack of observed diffusion of Mo can be attributed to the insufficient depth resolution of SIMS analysis. A slight broadening of the CN and S distribution profiles was found for these films, indicating a possible diffusion of organic molecules through the ZnO and MoO_3_. With these experiment results, one can conclude that thermal annealing on the cells induces intensive interaction between MoO_3_ and the organic photoactive layer, which condenses the PM6:Y6/MoO_3_ interface, on the other hand, and leads to the formation of Mo^5+^ by the chemical reduction Mo^6+^ with organic compounds. Attempts to detect the oxidized organic matter at the interface with mass spectrometry were made. However, no direct evidence of the formation of oxidized organic compounds was found, possibly due to a low concentration of the molecules. Nevertheless, the current experiment results confirmed the thermal-induced intermolecular interaction between MoO_3_ and PM6:Y6, ascribed to the main reason for the thermal-induced performance decay of the cells. For the first time, this work revealed the thermal-induced degradation pathway of NFA solar cells at the interface between MoO_3_ and photoactive layer.

### 2.4. Stability Improvement by Inserting a C_60_ Layer

Based on the experimental results described above, it can be concluded that the enhanced interfacial chemical interaction between PM6:Y6 and MoO_3_ is the main reason causing the performance degradation upon high-temperature thermal annealing. Inserting a thin blocking layer to avoid the interfacial contact of MoO_3_ and BHJ is expected to passivate the thermal-induced aging. Various insulators (including Al_2_O_3_, PVP, PEG, PDMS) were inserted between the PM6:Y6/MoO_3_ interface to impede the interfacial contact. Although device performance was not significantly influenced by inserting the isolating layer, the performance of these cells under thermal annealing was not stabilized (Tables S13–S16 in supporting information). These results indicated that the diffusion and the reduction MoO_3_ cannot be suppressed by simply inserting insulator layers.

**Figure 4:**
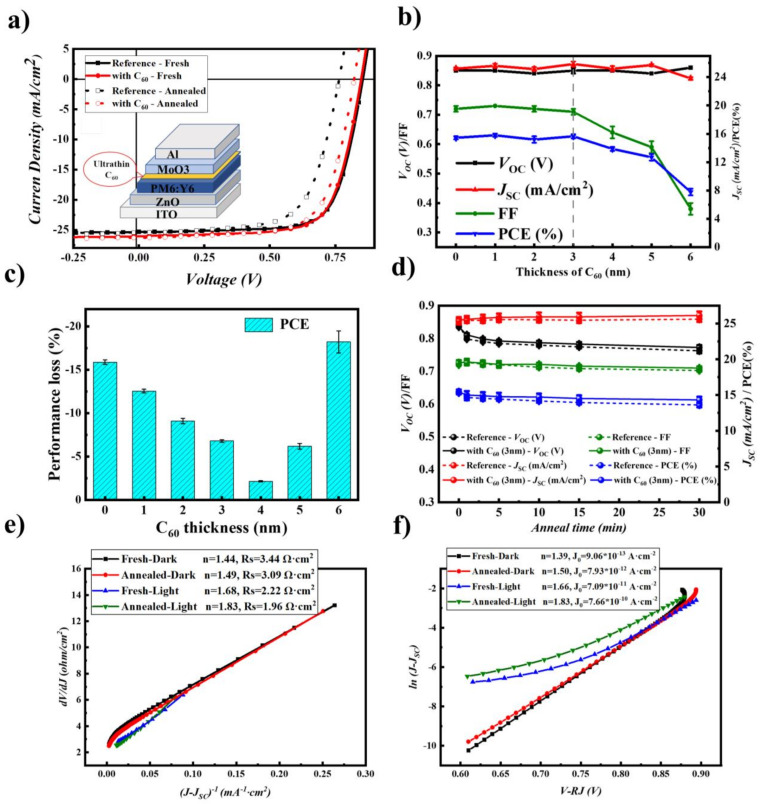
(**a**) *J* − *V* curves of reference and 3 nm-C_60_ modified cells before/after thermal annealing; (**b**) performance of the cells with different thicknesses of C_60_; (**c**) PCE losses of the cells with different C_60_ layer thickness upon thermal annealing at 150 °C for 5 min; (**d**) variation of cell performance upon different thermal annealing time for the 3 nm-C_60_ modified cells. The reference cell was included for comparison; (**e**) the fitted curve of d*V*/d*J* vs. (*J* − *J_SC_*)^−1^ for calculating *n* and *R_S_*; (**f**) the fitted curve of ln(*J* − *J_SC_*) vs. *V* − *R*_S_*J* for calculating *n* and *J*_0_.

Our previous works demonstrated that a thin C_60_ layer could suppress the interfacial reaction between the photoactive layer and MoO_3_ under illumination and improve the light stability of P3HT:PC_61_BM solar cells [36]. A thin C_60_ layer was then thermally evaporated on the top of the PM6:Y6 surface before the MoO_3_/Al electrode deposition. Figure 4a shows the *J* − *V* curves comparison of the cells without or with a 3 nm (optimized thickness) C_60_ layer. The full *J* − *V* curves of the cells with different layer thicknesses are shown in Figure S13. The correlation of device performance with the thickness of the C_60_ layer is shown in Figure 4b (see also Table S17 for the complete device performance of the cells). As seen here, the PCE of the cells increased slightly after the insertion of a thin C_60_ layer (15.65% for the 3 nm C_60_ and 15.44% for the reference devices). The improvement in device performance was mainly ascribed to the increase of short circuit current and fill factor, which can be ascribed to the enhanced charge selectivity effect of C_60_ at the anode interface [8,41]. Further increasing the layer thickness of C_60_, however, decreases the device performance by affecting the fill factor and short circuit current, causing pronounced S-shape *J* − *V* curves (Figure S13a), which can be ascribed to the hole-blocking nature of the C_60_ layer.

We then checked the thermal stability of the cells upon thermal annealing at 150 °C. Figure 4c shows the performance variation of the cells with different C_60_ layer thicknesses, and the detailed results are listed in Table S17 as well. Inserting the C_60_ layer can improve the thermal stability of the cells, in particular for the *V*_OC_ and FF, which were significantly slowed down with the increase of C_60_ layer thickness. The optimized device with 3 nm of C_60_ as the passivation layer showed the highest PCE of 14.58% after thermal annealing at 150 °C for 5 min, maintaining 93% of its initial PCE. We noted that the C_60_-modified devices also showed a *V*_OC_ loss under heated (from 0.848 V to 0.822 V), similar to that of the BHJ-heated devices (seen as Figure 2 Device B), suggesting such degradation may come from the ZnO/BHJ interface or other layers. Figure 4d compares the thermal stability of C_60_-inserted and reference devices for a longer time with device performance statistics. The C_60_-incorporated cells showed a high average PCE of 14.31%, corresponding to 93% of its initial performance (15.43%).

In contrast, the PCE of C_60_-free cells showed an average PCE of 13.61%, with an 11% loss of its initial value (15.31%). These results confirm that the C_60_ interlayer can significantly improve the thermal stability of the cells. Furthermore, it confirms that the PM6:Y6/MoO_3_ interfacial degradation is the main reason causing the performance decay upon thermal annealing. Figure 4e,f show the plots of *dV*/*dJ* vs. (*J* − *J_SC_)*^−1^ and ln (*J* − *J_SC_)* vs. *V* − *RsJ*, respectively. The series resistance *Rs*, ideality factor *n*, and the saturated dark current density *J*_0_ were then obtained according to Equations (2) and (3), and the detailed data are listed in Table S18. Unlike the C_60_ free cell, the cells with the C_60_ interlayer showed much less variation upon thermal annealing, confirming the stabilization effect of the C_60_ interlayer at the PM6:Y6/MoO_3_ interface.

## 3. Materials and Methods

### 3.1. Materials

ITO substrates are custom-made on glass with a strip width of 3 mm. PM6 (PBDB-T-2F) and Y6 (BTP-4F) were purchased from Solarmer Materials Inc., Beijing, China. 1-chloronaphthalene (CN) was purchased from sigma-Aldrich. Molybdenum (VI) oxide (MoO_3_) was purchased from Strem Chemicals Inc., TLtd, Newburyport, USA. C_60_ (99.5%) was purchased from Suzhou Dade Carbon Nano Technology Co. Ltd., Suzhou, China. Chloroform (CF, 99.80%) was purchased from Adamas-beta. All materials were used as received without further purification. ZnO nanoparticles solution was prepared through the reaction between KOH and Zn(OAc)_2_ in methanol as reported by Beek et al.

### 3.2. Organic Solar Cells Device Fabrication Methods

ITO substrates were sequentially cleaned by detergent, deionized water, acetone (twice) and isopropanol (twice) in an ultrasound cleaner, after which they were stored in isopropanol. Before use, the ITO glass sheets were blow-dried by N_2_ flow and then treated in a UV-ozone oven for 15 min. ZnO NPs solution (12 mg/mL in Methanol) was spin-coated on the ITO substrates at 2500 rpm for 60 s and then annealed at 130 °C for 10 min on a hot plate. The blend solution of PM6:Y6 with 0.5 vol% CN in chloroform was stirred for 2 h (the concentrations of PM6 and Y6 are 7 and 8.4 mg/mL, respectively). The PM6:Y6 photoactive layer film was fabricated by spinning coating the above solution at 2000 rpm for 30 s and the film was heated under 100 °C for 10 min. Finally, MoO_3_ (10 nm) and Al (100 nm) were deposited on the top of the active layer by vacuum thermal evaporation. The effective photovoltaic area for each device was 0.09 cm^2^.

### 3.3. Organic Solar Cells Performance Characterizations

The PV parameters of the devices (*V*_OC_, *J_SC_*, FF and PCE) were measured under a simulated AM 1.5G sunlight (Verasol-2, LED 3A Sun simulator, Newport) in a glove box filled with N_2_. Kithley 2400 was used as source meter. A homemade system recorded the external quantum efficiency (EQE) spectra. The probe light was from a 150 W tungsten halogen lamp (Osram 64610), modulated with a mechanical chopper before passing through the monochromator (Zolix, Omni-k300) to separate the certain wavelength. A stand silicon cell was tested as the reference to calibrate light intensity. The response was recorded as the voltage by an I-V converter (D&R-IV Converter, Suzhou D&R Instruments and Equipment Co., Ltd., Suzhou, China) with a lock-in amplifier (SR 830, Stanford Research Systems, Sunnyvale, CA, USA).

The thermal-induced degradation was performed by heating devices on a hot-plate in the glove box filled with N_2_. All the devices were tested as described above manually after a certain cooling time.

### 3.4. Instruments and Measurement

A PerkinElmer Lambada 750 was used to measure UV-Vis absorption spectra of films. The mass spectra of PM6/Y6 and MoO_3_ blends were measured with Matrix-Assisted Laser Desorption/Ionization Time-Of-Flight Mass Spectrometry (MALDI-TOF-MS). The AFM images were taken with a Dimension Icon. The XPS and UPS spectra were measured through PHI 5000 VersaProbe III. The SIMS data were tested through IONTOF TOF.SIMS 5–100.

## 4. Conclusions

In summary, we systematically investigated the thermal-induced degradation of the high-performance polymer:non-fullerene (PM6:Y6) solar cells with an inverted structure. The decay of *V*_OC_ and FF led to the degradation of device performance under thermal annealing. By pre-annealing on different half-cells, results demonstrated that thermal annealing-induced performance decay of PM6:Y6 cells is mostly originated from the decay at PM6:Y6/MoO_3_. SIMS, UV–Vis absorption, and XPS measurements were performed to confirm the intensive interaction at the PM6:Y6/MoO_3_ interface. The increased ideality factor and reduced built-in potential voltage confirmed by dark *J* – *V* curve analysis further confirmed the increased interfacial charge recombination after thermal annealing. Inserting a thin layer of C_60_ (3–4 nm) stabilized the cell performance upon thermal annealing, ascribed to efficiently blocking the contact of MoO_3_ and PM6:Y6 layer. The C_60_-incorporated cells (3 nm) showed a PCE degradation of 7% of their initial performance while C_60_-free cells showed an 11% loss of their initial value. The current work provides a comprehensive investigation of the thermal induced degradation mechanism of polymer:non-fullerene solar cell and a feasible and applicable interface modification method were proposed to suppressed performance degradation. For the first time, this work revealed the mechanism of thermal-induced interfacial degradation of NFA solar cells and explored an effective way to solve the problem, which is expected to promote the commercial application of organic solar cells.

## Data Availability

The supporting data are available in the Supplementary Materials.

## Supplementary Materials

The following supporting information can be downloaded at: https://www.mdpi.com/article/10.3390/molecules28196856/s1.

## Sample Availability

Not available.
