# Supplementary material for: Thermal-Induced Performance Decay of the State-of-the-Art Polymer: Non-Fullerene Solar Cells and the Method of Suppression
Source: Molecules. 2023 Sep 28;28(19):6856. doi: 10.3390/molecules28196856 (PMC10574091; doi:10.3390/molecules28196856)
Supplement: Supplementary file 1 [file molecules-28-06856-s001.zip › molecules-2601042-supplementary.pdf]

Supporting Information

Thermal-induced Performance Decay of the State-of-the-art Polymer: Non-fullerene Solar Cells and the Method of Suppression

Xingxing Qin [1,2], Xuelai Yu [1,3], Zerui Li [1], Jin Fang [1], Lingpeng Yan [1,4], Na Wu [1,*], Mathias Nyman [5],
Ronald Österbacka [1,5], Rong Huang [6], Zhiyun Li [6] and Chang-Qi Ma [1,3,*]

[1] i-Lab &Printed Electronics Research Center, Suzhou Institute of Nano-Tech and Nano-Bionics, Chinese Academy of Sciences (CAS), Suzhou 215123, China
[2] Nano Science and Technology Institute, University of Science and Technology of China, 166 Ren Ai Road, SEID SIP, Suzhou 215123, China
[3] School of Nano-Tech and Nano-Bionics, University of Science and Technology of China, 398 Jinzhai Road, Hefei 230026, China
[4] College of Materials Science and Engineering, Taiyuan University of Technology, Taiyuan 030024, China
[5] Physics and Center for Functional Materials, Faculty of Science and Technology, Åbo Akademi University, Henriksgatan 2, Turku 20500, Finland
[6] Vacuum Interconnected Nanotech Workstation, Suzhou Institute of Nano-Tech and Nano-Bionics, Chinese Academy of Sciences (CAS), 398 Ruoshui Road, SEID, SIP, Suzhou 215123, China
* Correspondence: nwu2022@sinano.ac.cn (N.W.); cqma2011@sinano.ac.cn (C.-Q.M.)

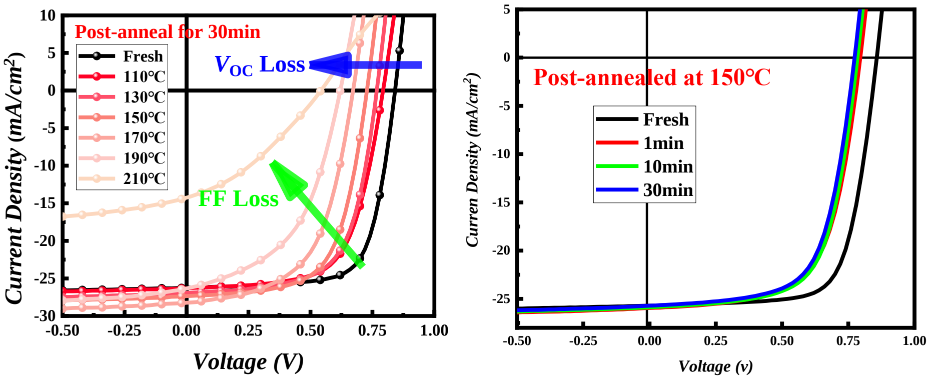

Figure S1. a) *J-V* curves of a representative cell annealed at different temperatures for 30 minutes. b) J-V curves of a representative cell annealed at 150°C for different times.

Table S1. Device performance of PM6:Y6 cells annealed at different temperatures for 30 minutes.

Entry	Annealing temperature	V_{OC} (*V*)	J_{SC} (*mA/cm*^{2})	FF	PCE (%)
1	Reference	0.835 ± 0.000	26.97 ± 0.51	0.69 ± 0.01	15.64 ± 0.32
2	70°C	0.815 ± 0.001	25.28 ± 0.51	0.68 ± 0.00	14.12 ± 0.14
3	80°C	0.832 ± 0.001	24.46 ± 0.59	0.70 ± 0.01	14.18 ± 0.19
4	90°C	0.834 ± 0.000	23.68 ± 0.22	0.69 ± 0.00	13.59 ± 0.12
5	100°C	0.838 ± 0.001	23.74 ± 1.09	0.66 ± 0.02	12.70 ± 0.28
6	110°C	0.774 ± 0.001	26.75 ± 0.30	0.66 ± 0.01	13.58 ± 0.33
7	130°C	0.761 ± 0.001	27.36 ± 0.43	0.64 ± 0.01	13.36 ± 0.13
8	150°C	0.728 ± 0.000	27.54 ± 0.30	0.63 ± 0.01	12.67 ± 0.14
9	170°C	0.675 ± 0.000	28.01 ± 0.20	0.49 ± 0.00	10.75 ± 0.15
10	190°C	0.618 ± 0.000	26.69 ± 0.42	0.42 ± 0.01	8.06 ± 0.17
11	200°C	0.561 ± 0.002	21.52 ± 0.64	0.42 ± 0.01	5.09 ± 0.31
12	210°C	0.502 ± 0.005	13.08 ± 1.61	0.33 ± 0.01	2.21 ± 0.58

Table S2. Device performance of PM6:Y6 cells upon thermal annealing at 150℃.

Entry	Annealing time	V_{OC} (*V*)	J_{SC} (*mA/cm*^{2})	FF	PCE (%)
13	w/o	0.841 ± 0.001	26.66 ± 0.65	0.70 ± 0.00	15.76 ± 0.43
14	1 min	0.767 ± 0.003	26.14 ± 0.66	0.61 ± 0.00	12.44 ± 0.35
15	3 min	0.765 ± 0.004	25.85 ± 0.09	0.61 ± 0.00	12.27 ± 0.06
16	5 min	0.765 ± 0.002	26.23 ± 0.74	0.62 ± 0.00	12.44 ± 0.38
17	10 min	0.762 ± 0.003	25.96 ± 0.88	0.62 ± 0.00	12.20 ± 0.32
18	30 min	0.756 ± 0.006	25.68 ± 0.70	0.61 ± 0.00	11.72 ± 0.32

Table S3. Fitted device performance of PM6:Y6 standard cells upon light and dark.

Device	Illumination condition	J_0 *(A/cm²)*	*Rs* (*Ω/cm²*)	Ideality factor *n*
Fresh	light	1.61×10^{-10}	2.45	1.75
Annealed	light	1.67×10^{-8}	2.17	1.99
Fresh	dark	2.44×10^{-13}	3.01	1.29
Annealed	dark	4.66×10^{-11}	2.28	1.49

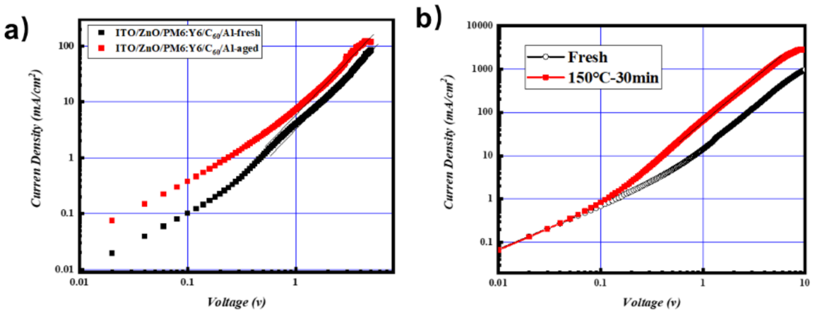

Figure S2. a) *J*-*V* curves of electron-only devices with a structure of ITO/ZnO/PM6:Y6/C_{60}/Al. b) *J*-*V* curves of hole-only devices with a structure of ITO/PEDOT:PSS/PM6:Y6/MoO_3/Al.

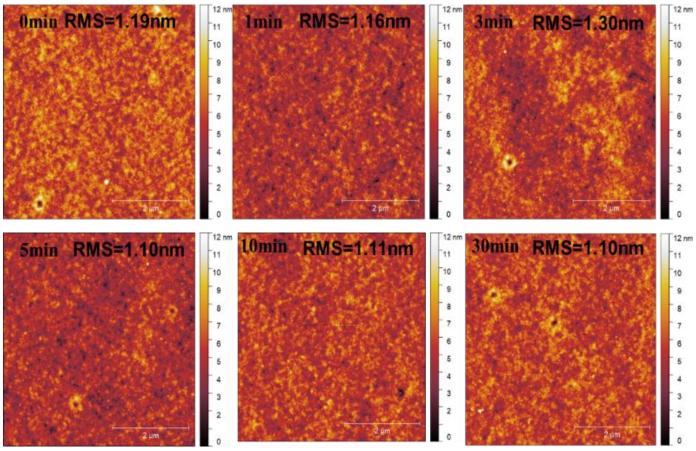

Figure S3. AFM image of BHJ/MoO_3 surface before and after annealing.

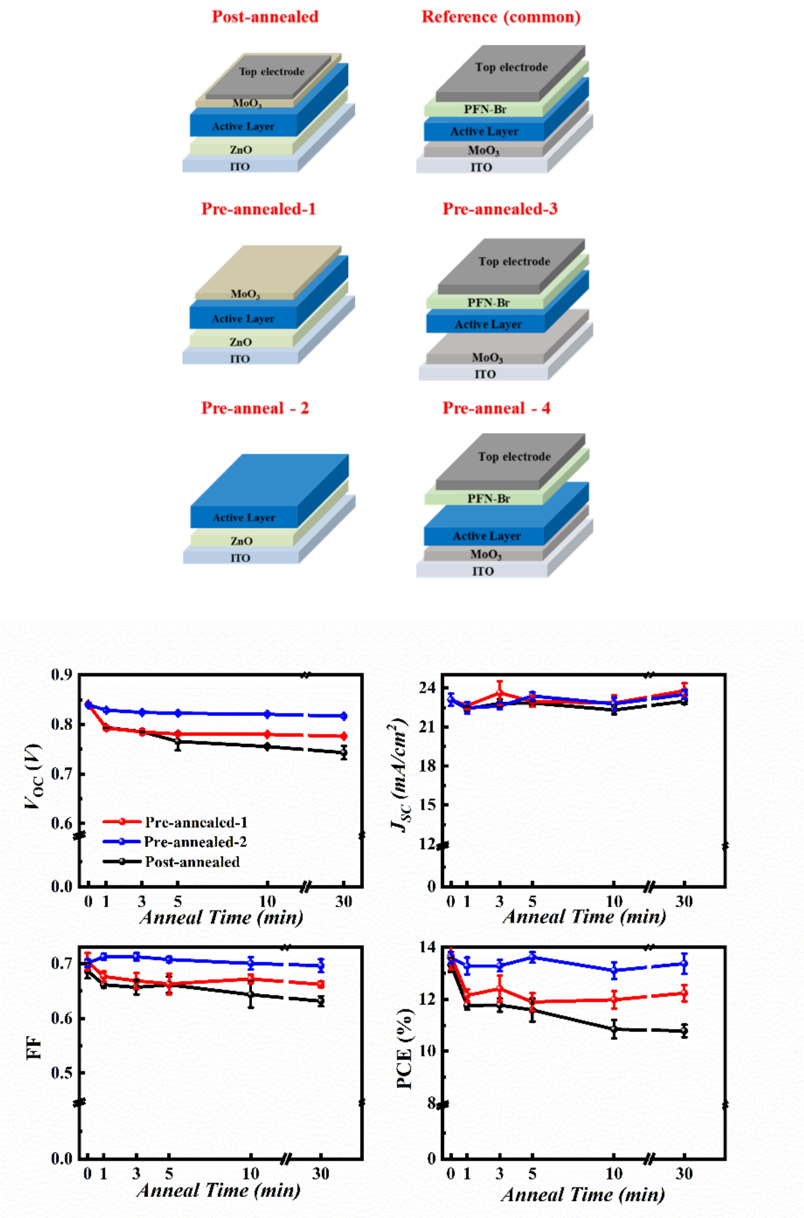

Figure S4. a) Structure of devices designed to severally annealing. b) Performance change with annealing time at 150°C of the three inverted devices annealed.

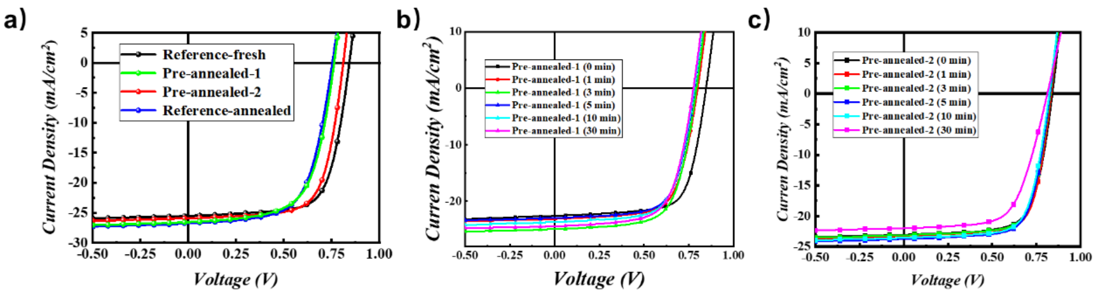

Figure S5. a) *J-V* curves of the three inverted devices annealed at 150°C for 10 minutes; b) *J-V* curves of devices annealed as pre-annealing ZnO/BHJ at 150°C for different time; c) *J-V* curves of devices annealed as pre-annealing BHJ/MoO_3 at 150°C for different time.

Table S4. Performance of devices pre-annealed at BHJ/MoO_3 interface (Device B).

Pre-annealed-1	V_{OC} (V)	J_{SC} (mA/cm^2)	FF	PCE (%)
0 min	0.841±0.003	23.09±0.47	0.70±0.02	13.675±0.539
30 min	0.776±0.001	23.79±0.57	0.66±0.01	12.236±0.311

Table S5. Performance of devices pre-annealed at ZnO/BHJ interface (Device C).

Pre-annealed-2	V_{OC} (V)	J_{SC} (mA/cm^2)	FF	PCE (%)
0 min	0.839±0.003	23.09±0.47	0.70±0.01	13.599±0.223
30 min	0.817±0.003	23.50±0.35	0.70±0.01	13.371±0.388

Table S6. Performance of post-annealed devices (Device A)

	V_{OC} (V)	J_{SC} (mA/cm^2)	FF	PCE (%)
0 min	0.842±0.003	23.09±0.58	0.69±0.01	13.325±0.272
30 min	0.743±0.013	22.98±0.22	0.63±0.01	10.785±0.239

Table S7. Device performance of common PM6:Y6 cells annealed at different stages.

Device	V_{OC} (V)	J_{SC} (mA/cm^2)	FF	PCE (%)	△PCE/PCE_0
Reference	0.833±0.000	23.49±0.37	0.68±0.00	13.26±0.24	--
Pre-annealed-3-Device D-1 min	0.832±0.000	24.23±0.34	0.69±0.00	13.87±0.26	3.9%
Pre-annealed-3-Device D-10 min	0.832±0.010	24.03±0.19	0.69±0.01	13.76±0.16	2.4%
Pre-annealed-4-Device E-1 min	0.804±0.000	23.29±0.37	0.65±0.00	12.11±0.24	-8.4%
Pre-annealed-4-Device E-10 min	0.781±0.000	23.06±0.38	0.63±0.01	11.33±0.14	-14.2%

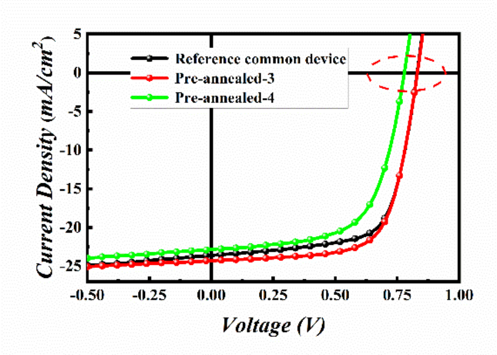

Figure S6. *J-V* curves of the three common devices (shown as Figure S4) annealed at 150°C for 10 minutes

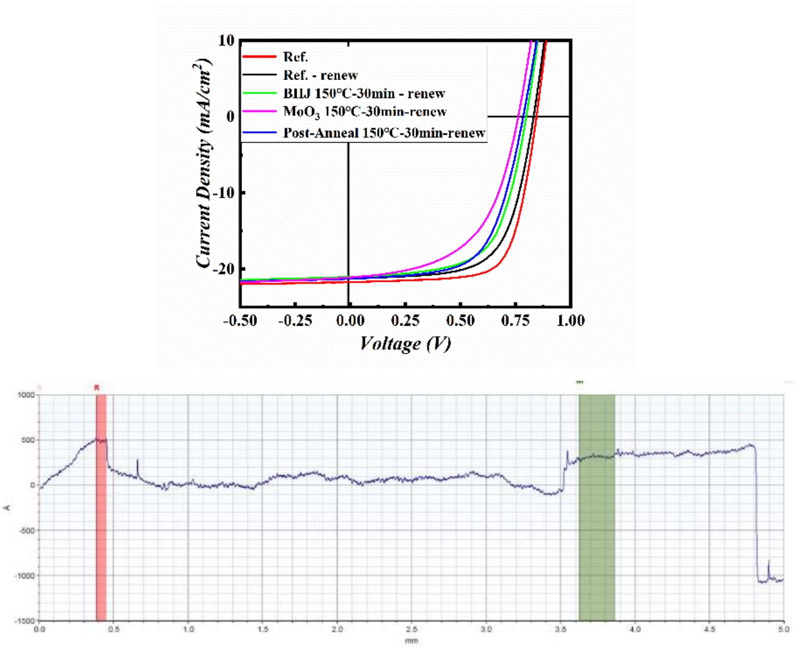

Figure S7. *J-V* curves of devices with renewed top electrode and profile of interface after peeling of top electrode.

Table S8. Device performance of devices with renewed top electrode.

Devices		V_{OC} (*V*)	J_{SC} (*mA/cm*^{2})	FF	PCE (%)
Reference	Pristine cell	0.841	21.16	0.69	12.21
	Renewed Al electrode	0.840	20.72	0.67	11.62
Pre-annealed at BHJ/MoO_3 interface	Pristine cell	0.823	21.65	0.68	11.89
	Renewed Al electrode	0.809	20.85	0.64	10.75
Pre-annealed at ZnO/BHJ interface	Pristine cell	0.782	21.84	0.63	10.74
	Renewed Al electrode	0.790	20.86	0.64	10.49
Post-annealed device	Pristine cell	0.756	21.45	0.54	8.61
	Renewed Al electrode	0.786	20.33	0.62	9.84

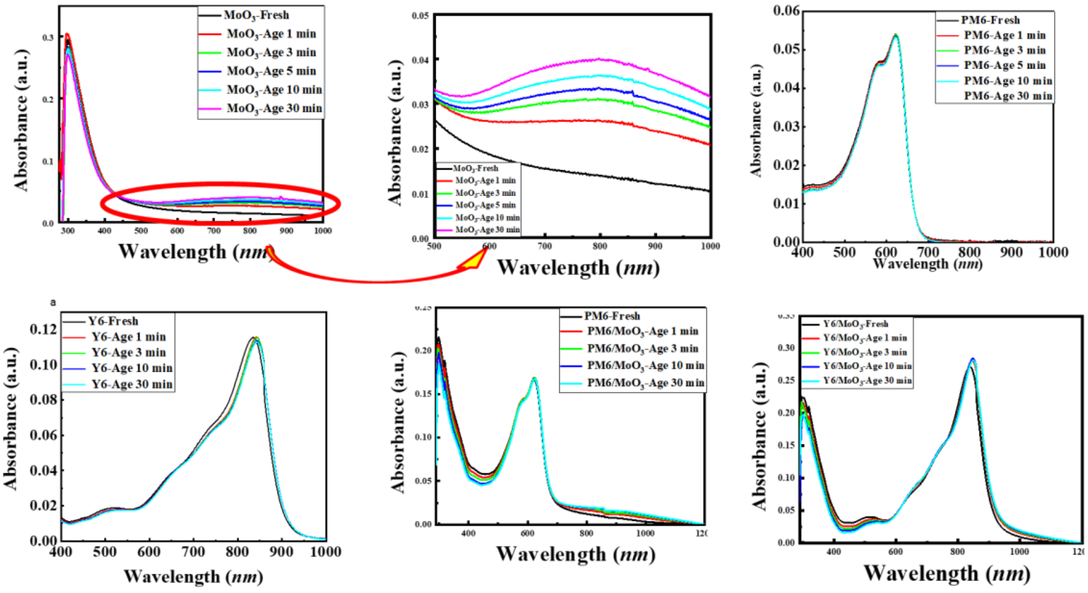

Figure S8. UV-vis spectra of MoO_3, PM6/MoO_3, Y6/MoO_3 films before and after annealing.

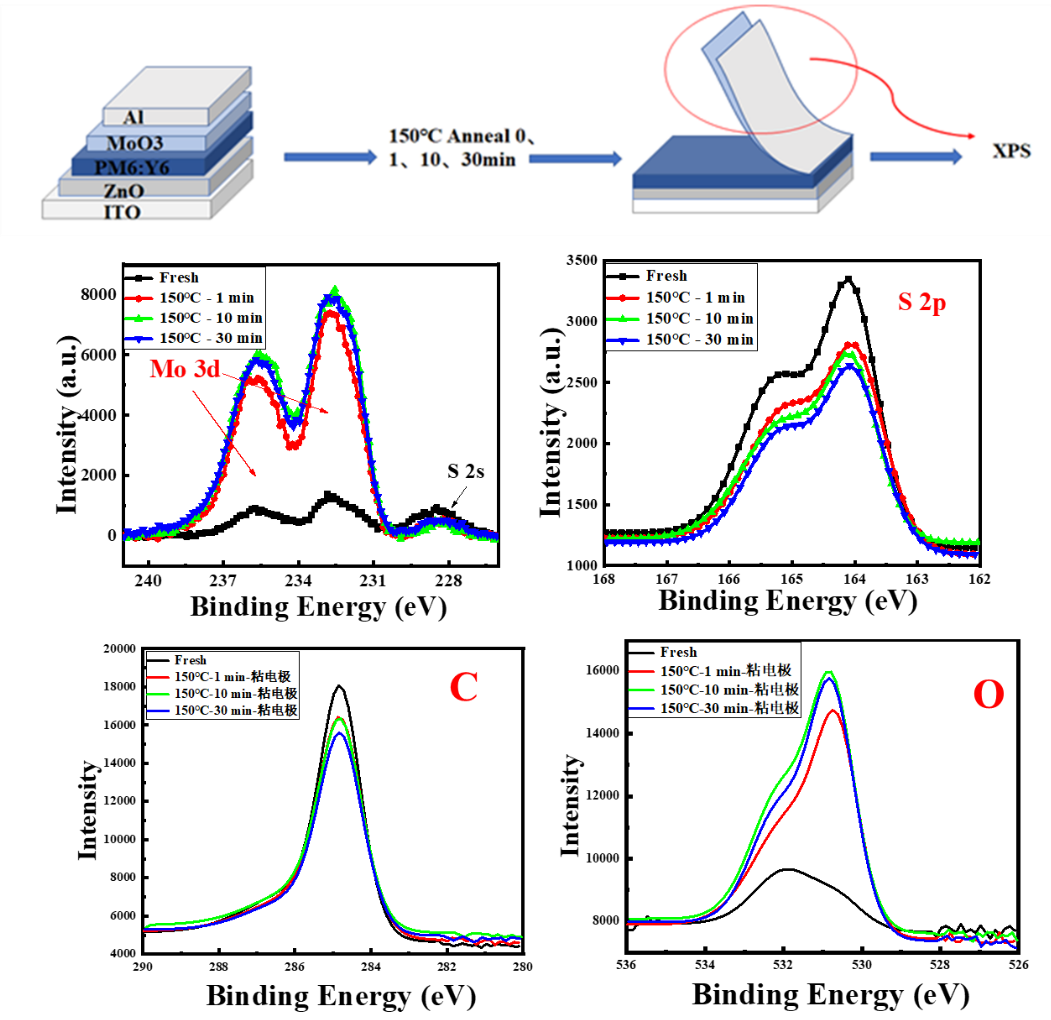

Figure S9. Diagram of XPS samples and XPS spectra of BHJ/MoO_3 interface, Mo 3d, S 2p, C 1s, O 1s, respectively.

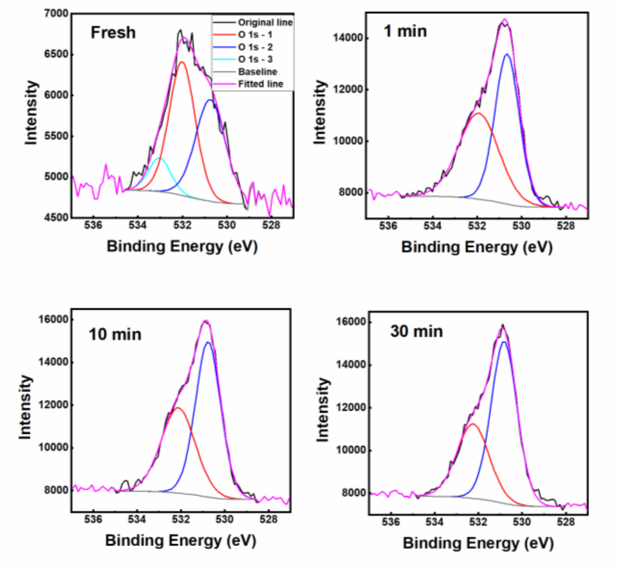

Figure S10. O 1s XPS spectra of BHJ/MoO_3 interface with different anneal time.

Table S9. Proportion of O in Mo of BHJ/MoO_3 interface before and after annealing.

		O 1s 1	O 1s 2	O 1s 3
Fresh	Peak position (eV)	532.01	530.74	533.03
	Proportion (%)	47.97	41.81	10.22
150°C 1 min	Peak position (eV)	531.93	530.65	--
	Proportion (%)	48.54	51.46	--
150°C 10 min	Peak position (eV)	532.12	530.76	--
	Proportion (%)	42.56	57.44	--
150°C 30 min	Peak position (eV)	532.24	530.82	--
	Proportion (%)	36.23	63.77	--

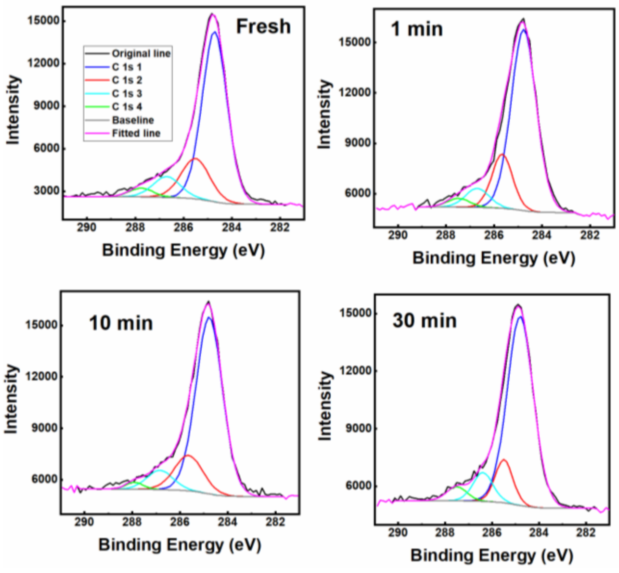

Figure S11. C 1s XPS spectra of BHJ/MoO_3 interface with different anneal time.

Table S10. Proportion of C in Mo of BHJ/MoO3 interface before and after annealing.

		C 1s 1	C 1s 2	C 1s 3	C 1s 4
Fresh	Peak position (eV)	284.78	285.51	286.70	287.75
	Proportion (%)	68.74	18.63	9.27	3.36
150℃ 1 min	Peak position (eV)	284.76	285.66	286.70	287.48
	Proportion (%)	71.94	17.69	7.21	3.17
150℃ 10 min	Peak position (eV)	284.77	285.64	286.84	287.90
	Proportion (%)	74.27	15.93	7.96	1.85
150℃ 30 min	Peak position (eV)	284.79	285.49	286.39	287.50
	Proportion (%)	75.31	11.65	8.72	4.33

Table S11. Proportion of Mo^{5+} in Mo of BHJ/MoO3 interface before and after annealing.

	Annealing time	Mo^{6+}		Mo^{5+}		Mo^{5+}/Mo (%)
S1	0	2104.8	1410.2	217.4	145.9	9.38
S2	1 min	14908.8	10135.0	2294.6	1537.4	13.27
S3	10 min	16891.7	11317.5	3773.6	2515.8	18.23
S4	30 min	15484.8	10323.2	3899.1	2612.4	20.15

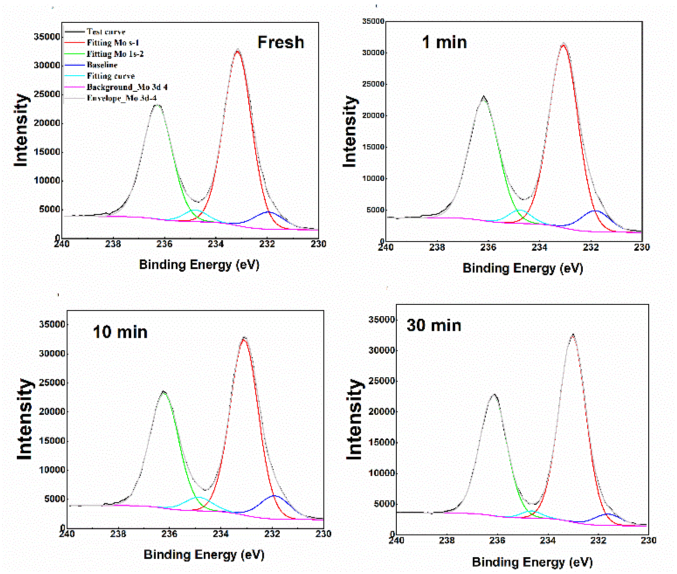

Figure S12. Mo 3d XPS spectra of MoO3 interface with different anneal time.

Table S12. Proportion of Mo^{5+} in Mo of MoO_3 before and after annealing.

	Annealing time	Mo^{6+}		Mo^{5+}		Mo^{5+}/Mo(%)
S1	0	42690.5	28602.7	4058.6	2719.3	8.68
S2	1 min	41989.2	28132.8	4979.0	3335.9	10.6
S3	10 min	42236.7	28298.6	5299.4	3550.6	11.15
S4	30 min	41494.5	27801.3	4662.2	3123.7	10.10

Table S13. Performance change of Al_2O_3 modified devices before and after thermal aging.

Concentration	V_{OC} *(V)*	J_{SC} *(mA/cm²)*	FF	PCE (%)
		Fresh		
w/o	0.832±0.006	20.54±0.12	0.70±0.02	12.00±0.54
1/100 c*	0.808±0.002	18.23±0.15	0.70±0.01	10.00±0.34
1/50 c	0.810±0.002	18.20±0.11	0.57±0.01	8.41±0.13
1/25 c	0.800±0.005	1.29±0.12	0.24±0.01	0.24±0.02
		Aged at 150 °C for 30 min		
w/o	0.766±0.002	20.45±0.13	0.68±0.01	10.61±0.12
1/100 c	0.715±0.006	18.56±0.17	0.60±0.00	7.93±0.07
1/50 c	0.737±0.005	18.79±0.13	0.51±0.01	7.07±0.08
1/25 c	0.727±0.011	2.46±0.23	0.22±0.01	0.34±0.04

*c: The concentration of purchased Al_2O_3 solution.

Table S14. Performance change of PVP modified devices before and after thermal aging.

Concentration	V_{OC} *(V)*	J_{SC} *(mA/cm²)*	FF	PCE (%)
		Fresh		
w/o	0.835±0.003	18.56±0.46	0.70±0.02	10.91±0.35
Methanol	0.833±0.003	18.68±0.25	0.71±0.00	11.10±0.21
0.5 mg/mL	0.752±0.023	18.68±0.43	0.59±0.02	8.24±0.68
		Aged at 150°C for 30 min		
w/o	0.727±0.023	18.83±0.43	0.65±0.02	8.92±0.66
Methanol	0.717±0.023	19.01±0.21	0.68±0.02	8.85±0.57
0.5 mg/mL	0.542±0.007	19.05±0.41	0.50±0.01	5.17±0.23

Table S15. Performance change of PEG modified devices before and after thermal aging.

Concentration	V_{OC} (V)	J_{SC} (mA/cm^2)	FF	PCE (%)
		Fresh		
w/o	0.851±0.002	21.56±0.18	0.74±0.01	13.57±0.14
Methanol	0.849±0.002	21.44±0.17	0.74±0.00	13.49±0.15
0.1 mg/mL	0.847±0.002	21.70±0.19	0.74±0.01	13.59±0.13
0.25 mg/mL	0.848±0.003	21.38±0.27	0.73±0.01	13.33±0.25
0.50 mg/mL	0.848±0.002	21.62±0.30	0.73±0.01	13.38±0.26
5.0 mg/mL	0.832±0.002	18.98±0.24	0.68±0.00	10.79±0.15
		Aged at 150°C for 30 min		
w/o	0.730±0.012	21.97±0.21	0.66±0.01	10.62±0.26
Methanol	0.726±0.010	21.94±0.16	0.66±0.01	10.46±0.22
0.1 mg/mL	0.732±0.006	22.03±0.42	0.66±0.01	10.67±0.39
0.25 mg/mL	0.723±0.012	21.67±0.30	0.64±0.02	10.06±0.36
0.50 mg/mL	0.722±0.004	21.99±0.22	0.65±0.00	10.39±0.13
5.0 mg/mL	0.38±0.01	16.07±1.28	0.44±0.01	2.68.±0.20

Table S16. Performance change of PDMS modified devices before and after thermal aging.

Concentration	V_{OC} (V)	J_{SC} (mA/cm^2)	FF	PCE (%)
		Fresh		
w/o	0.842±0.003	19.79±0.11	0.69±0.00	11.56±0.12
n-butanol	0.841±0.002	20.17±0.41	0.69±0.02	11.78±0.34
1 mg/mL	0.839±0.008	20.56±0.34	0.69±0.01	12.04±0.20
2.5 mg/mL	0.822±0.008	19.45±0.33	0.58±0.01	9.31±0.20
5.0 mg/mL	0.840±0.002	18.50±0.27	0.64±0.18	9.88±0.44
		Aged at 150°C for 30 min		
w/o	0.752±0.002	20.30±0.21	0.56±0.01	8.61±0.15
n-butanol	0.753±0.008	20.18±0.44	0.59±0.02	8.86±0.20
1 mg/mL	0.749±0.007	20.43±0.40	0.58±0.02	8.87±0.22
2.5 mg/mL	0.741±0.006	19.74±0.44	0.51±0.16	7.42±0.23
5.0 mg/mL	--	--	--	--

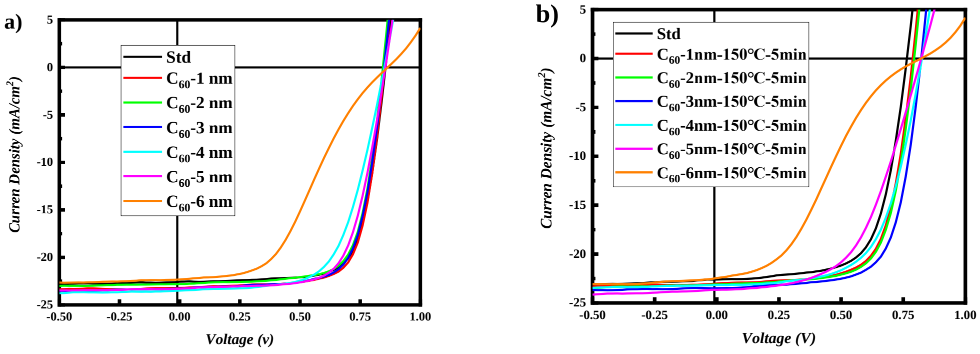

Figure S13. a) *J-V* curves of C_{60} inserting devices with different C_{60} thicknesses; b) J-V curves of C_{60} inserting devices after post-annealing.

Table S17. Performance change of C_{60} modified devices before and after thermal aging.

Thickness (nm)	V_{OC} *(V)*	J_{SC} *(mA/cm²)*	FF	PCE (%)	ΔPCE/ PCE_0(%)
			Fresh		
0	0.847±0.003	25.21±0.16	0.72±0.01	15.44±0.22	
1	0.849±0.004	25.60±0.29	0.73±0.00	15.80±0.23	
2	0.844±0.003	25.14±0.28	0.72±0.01	15.19±0.46	
3	0.848±0.003	25.85±0.36	0.71±0.01	15.65±0.29	
4	0.851±0.002	25.20±0.35	0.64±0.02	13.87±0.28	
5	0.852±0.001	25.69±0.15	0.59±0.02	12.69±0.46	
6	0.856±0.003	23.81±0.19	0.38±0.02	7.87±0.47	
			Aged at 150°C for 5 min		
0	0.764±0.003	25.25±0.17	0.67±0.00	12.99±0.09	-15.87
1	0.789±0.005	25.17±0.24	0.70±0.00	13.81±0.12	--12.59
2	0.789±0.006	25.38±0.25	0.69±0.00	13.81±0.23	--9.08
3	0.822±0.001	25.67±0.16	0.69±0.00	14.58±0.09	-6.84
4	0.825±0.001	25.65±0.20	0.64±0.02	13.57±0.33	-2.16
5	0.820±0.001	25.92±0.18	0.56±0.02	11.91±0.48	-6.15
6	0.822±0.001	25.11±0.31	0.31±0.01	6.43±0.23	-18.30

Table S18. Fitted device performance of PM6:Y6 standard cells upon light and dark.

Device	state	J_0 *(A/cm²)*	*R*s (*ohm/cm²*)	Ideality factor *n*
Fresh	light	7.29×10^{-11}	2.22	1.68
Annealed	light	7.66×10^{-10}	1.96	1.83
Fresh	dark	9.06×10^{-13}	3.44	1.44
Annealed	dark	7.93×10^{-12}	3.09	1.49
